# Complementary action of granulocyte macrophage colony-stimulating factor and interleukin-17A induces interleukin-23, receptor activator of nuclear factor-κB ligand, and matrix metalloproteinases and drives bone and cartilage pathology in experimental arthritis: rationale for combination therapy in rheumatoid arthritis

**DOI:** 10.1186/s13075-015-0683-5

**Published:** 2015-06-17

**Authors:** Annemarie E. M. van Nieuwenhuijze, Fons A. van de Loo, Birgitte Walgreen, Miranda Bennink, Monique Helsen, Liduine van den Bersselaar, Ian P. Wicks, Wim B. van den Berg, Marije I. Koenders

**Affiliations:** Experimental Rheumatology, Radboud University Medical Centre, Route 272, Geert Grooteplein 28, 6525 GA Nijmegen, The Netherlands; Reid Rheumatology Laboratory, The Walter and Eliza Hall Institute of Medical Research, 1G Royal Parade, Parkville 3050, Melbourne, Australia; Autoimmune Genetics Laboratory, Vlaams Instituut voor Biotechnologie (VIB), and Department of Microbiology and Immunology, University of Leuven, Campus Gasthuisberg, Herestraat 49, Leuven, 3000 Belgium

## Abstract

**Introduction:**

Type 17 T helper cells and interleukin (IL)-17 play important roles in the pathogenesis of human and murine arthritis. Although there is a clear link between IL-17 and granulocyte macrophage colony-stimulating factor (GM-CSF) in the inflammatory cascade, details about their interaction in arthritic synovial joints are unclear. In view of the introduction of GM-CSF and IL-17 inhibitors to the clinic, we studied how IL-17 and GM-CSF orchestrate the local production of inflammatory mediators during experimental arthritis.

**Methods:**

To allow detection of additive, complementary or synergistic effects of IL-17 and GM-CSF, we used two opposing experimental approaches: treatment of arthritic mice with neutralising antibodies to IL-17 and GM-CSF and local overexpression of these cytokines in naive synovial joints. Mice were treated for 2 weeks with antibodies against IL-17 and/or GM-CSF after onset of collagen-induced arthritis. Naive mice were injected intraarticularly with adenoviral vectors for IL-17 and/or GM-CSF, resulting in local overexpression. Joint inflammation was monitored by macroscopic scoring, X-rays and histology. Joint washouts, synovial cell and lymph node cultures were analysed for cytokines, chemokines and inflammatory mediators by Luminex analysis, flow cytometry and quantitative polymerase chain reaction.

**Results:**

Combined therapeutic anti-IL-17 and anti-GM-CSF ameliorated arthritis progression, and joint damage was dramatically reduced compared with treatment with anti-IL-17 or anti-GM-CSF alone. Anti-IL-17 specifically reduced synovial IL-23 transcription, whereas anti-GM-CSF reduced transcription of matrix metalloproteinases (MMPs) and receptor activator of nuclear factor κB ligand (RANKL). Overexpression of IL-17 or GM-CSF in naive knee joints elicited extensive inflammatory infiltrate, cartilage damage and bone destruction. Combined overexpression revealed additive and synergistic effects on the production of MMPs, RANKL and IL-23 in the synovium and led to complete destruction of the joint structure within 7 days.

**Conclusions:**

IL-17 and GM-CSF differentially mediate the inflammatory process in arthritic joints and show complementary and local additive effects. Combined blockade in arthritic mice reduced joint damage not only by direct inhibition of IL-17 and GM-CSF but also by indirect inhibition of IL-23 and RANKL. Our results provide a rationale for combination therapy in autoinflammatory conditions, especially for patients who do not fully respond to inhibition of the separate cytokines.

## Introduction

Rheumatoid arthritis (RA) is a systemic disease characterized by chronic inflammation of synovial joints. The aetiology of RA remains unclear, but the pathogenesis has been studied extensively in patients as well as in animal models [1, 2]. In RA, inflammatory cells such as neutrophils, natural killer cells and T and B lymphocytes infiltrate the synovial membrane, contributing to bone and cartilage degradation. Of the T cells in the inflamed synovium, CD4^+^ T cells are the most abundant. CD4^+^ T cells can differentiate into type 1 T helper (Th1), Th2, Th17 or regulatory T cells, depending on the local cytokine milieu [3]. Originally, RA was classified as a Th1-mediated disease, but it is now well established that Th17 cells play a crucial role in RA pathogenesis (reviewed in [4]).

Th17 cells are important mediators of inflammatory disease and are identified by the production of interleukin (IL)-17 [5, 6]. Th17 cells are the main pathogenic cell type in many models of autoimmunity, including experimental autoimmune encephalomyelitis (EAE) [7], acute inflammatory arthritis [methylated bovine serum albumin (mBSA)/IL-1β] arthritis) [8] and collagen-induced arthritis (CIA) [9]. Pathogenic Th17 cells have also been identified in inflamed synovia of patients with RA [10]. Neutralisation of IL-17 during CIA showed that IL-17 plays a direct and indirect role in joint inflammation and joint destruction [11, 12]. IL-17 receptor–deficient mice show reduced sensitivity to inflammation [13].

Recent data illustrate that granulocyte macrophage colony-stimulating factor (GM-CSF) plays a crucial role in mediating T cell function in inflammatory conditions. IL-23 is required for functional maturation and pathogenicity of Th17 cells via RAR-related orphan receptor γt (RORγt)-mediated induction of GM-CSF production [14–17]. GM-CSF in turn also induces IL-23 production by dendritic cells (DCs) via CC chemokine receptor 4 (CCR4), thereby creating a positive feedback loop for the production of these two cytokines [18]. Interestingly, GM-CSF-deficient mice are resistant to many Th17-dependent models of autoimmune disease, including CIA, mBSA/IL-1β, EAE and experimental autoimmune myocarditis [8, 19–21], and GM-CSF-deficient Th17 cells are unable to induce EAE upon adoptive transfer [16]. GM-CSF-producing T cells were detected in synovial fluid taken from patients with juvenile idiopathic arthritis, and Th17 cells from these patients rapidly upregulated GM-CSF production in culture [22]. In addition, NF-κB1-deficient mice, which have a relatively selective defect in GM-CSF production by T cells, were protected from acute inflammatory arthritis and peritonitis [23]. Mice that overexpress GM-CSF specifically in T cells have increased Th17-related cytokines in the serum [24].

Although there is no doubt that both IL-17 and GM-CSF play indispensable roles in the pathogenesis of RA, the question remains what the interplay is between these cytokines. In the present study, we used two opposing approaches to address this question: (1) by combined neutralisation of GM-CSF and IL-17 during established CIA and (2) by combined local adenoviral overexpression of GM-CSF and IL-17 in the knee joint. Our results provide a rationale for combined neutralisation of GM-CSF and IL-17 in autoinflammatory conditions such as RA.

## Methods

### Mice

Eight- to ten-week-old male DBA/1 J mice (Janvier Labs, Saint Berthevin, France) were allowed to acclimatize for a minimum of 7 days in the Central Animal Laboratory of the Radboud University Medical Centre. The animals were kept in conventional filter-top cages (for the neutralisation experiment) or in specialised individually ventilated cages (for the adenoviral overexpression experiment). A maximum of 11 animals were kept in each cage. Food and water was provided ad libitum, and mice were subjected to 12-hour cycles of light and darkness.

All animal experiments were approved by the ethics committee of Radboud University Nijmegen (approval number 2013–042).

### Induction of arthritis

CIA was induced as previously described [25]. Joint inflammation was scored macroscopically in each paw using a scale of 0–2, where 0 = not inflamed, 1 = mild, 1.5 = marked and 2 = severe. Scoring was performed by a researcher blinded to the experimental groups. From day 21, mice were scored three times weekly and randomly divided over the treatment groups once the arthritis score reached between 0.25 and 1.25.

### Antibody treatment

Mice received intraperitoneal injections of anti-IL-17 (100 μg/mouse, clone MAB421; R&D Systems, Minneapolis, MN, USA), anti-GM-CSF (200 μg/mouse, clone 22E9; kind gift from Micromet AG, München, Germany), or isotype control antibody [300 μg/mouse, rat immunoglobulin G (IgG)] three times weekly from the onset of disease. The total duration of treatment for each mouse was 14 days.

### Adenoviral vectors

The adenoviral expression vector for murine IL-17A (Ad-IL-17) was kindly provided by Dr. J. K. Kolls (Children’s Hospital of Pittsburgh, Pittsburgh, PA, USA). The adenoviral expression vector for murine GM-CSF (Ad-GM-CSF) was provided by RIKEN BioResource Centre (Tsukuba, Japan), which is participating in the National BioResource Project of the MEXT (Ministry of Education, Culture, Sports, Science, and Technology, Tokyo, Japan). An adenoviral expression vector for luciferase (Ad-LUC) generated in-house was used as a control vector throughout the study. Virus titres [in focus forming units (ffu) per millilitre] were determined by performing focus forming assays as described previously [26].

### Intraarticular injection of adenoviral vectors

Mice received intraarticular injections of 6 μl of virus suspension in phosphate-buffered saline. For the groups treated with Ad-GM-CSF or Ad-IL-17 alone, 0.5 × 10^7^ ffu of the respective vectors was injected, supplemented with 0.5 × 10^7^ ffu of the control vector, Ad-LUC. For the group receiving both Ad-GM-CSF and Ad-IL-17, 0.5 × 10^7^ ffu of each vector was injected. Knee joints were harvested at 4 h and days 1, 4 and 7 after injection for analysis.

### RNA purification and quantitative real-time PCR

mRNA from synovial tissue was purified using TRIzol reagent (Sigma-Aldrich, St. Louis, MO, USA) and MagNA Lyser beads (Roche Diagnostics, Mannheim, Germany) according to the manufacturers’ instructions. cDNA was obtained by reverse transcription. Quantitative real-time PCR (qPCR) was performed using the ABI PRISM 7000 Sequence Detection System (Applied Biosystems, Foster City, CA, USA) for quantification with SYBR Green (Molecular Probes; Life Technologies, Carlsbad, CA, USA) and melting curve analysis. Fold increases of mRNA transcripts were calculated as follows: ΔCt = Ct (gene of interest) − Ct (glyceraldehyde 3-phosphate dehydrogenase), ΔΔCt = ΔCt sample − average ΔCt control group, and fold difference = 2^−ΔΔCt^.

### Patella washouts

Patellae, including attached synovial tissue, were removed from the knee joints and cultured in 200 μl of RPMI medium supplemented with 10 % foetal bovine serum for 1 h at room temperature. Supernatants were harvested for cytokine measurement.

### Flow cytometry

Single-cell suspensions from synovial tissue were prepared as described previously [27]. Synovial cells were stimulated in the short term (3–4 h) with phorbol 12-myristate 13-acetate (50 ng/mL) and ionomycin (1 μg/ml) before analysis to allow recovery of the cells after tissue digestion. Antibodies used were anti-CD4 Alexa Fluor 700 (clone GK1.5) and anti-CD8-PerCp (clone 53–6.7) (both from BioLegend, San Diego, CA, USA). All samples were measured on a CyAn ADP instrument (Beckman Coulter, Brea, CA, USA) and analysed using FlowJo software (FlowJo, Ashland, OR, USA).

### Measurement of cytokines and chemokines

Cytokines and chemokines were measured by Luminex technology according to the manufacturer’s instructions (EMD Millipore, Billerica, MA, USA). The results were analysed using BioPlex Manager 4 (Bio-Rad Laboratories, Hercules, CA, USA).

### Measurement of collagen-specific IgG in serum

Anti-collagen IgG1 and IgG2a antibody titres against bovine type II collagen (CII) were determined by enzyme-linked immunosorbent assay as described previously [11].

### X-ray analysis of ankle joints

X-rays from ankle joints were taken using a Faxitron MX20 instrument (Faxitron Bioptics, Tucson, AZ, USA) and analysed using Faxitron software.

### Histology

Joints were fixed for 4 days in 10 % formalin and decalcified in 5 % formic acid, and then they were embedded in paraffin. Seven-micrometre sections were stained with haematoxylin and eosin (H&E) or Safranin O (SafO). Inflammation, chondrocyte death, bone destruction, cartilage damage and proteoglycan (PG) depletion were scored on a scale of 0–3 by a researcher blinded to the experimental groups.

### Statistical analysis

Statistical significance was calculated using the Prism 6 software package (GraphPad Software, La Jolla, CA, USA) by one- or two-way analysis of variance, followed by Bonferroni’s multiple-comparisons test. *p* values <0.05 were considered significant.

## Results

### Combined neutralisation of GM-CSF and IL-17 blocks progression of CIA

**Fig. 1:**
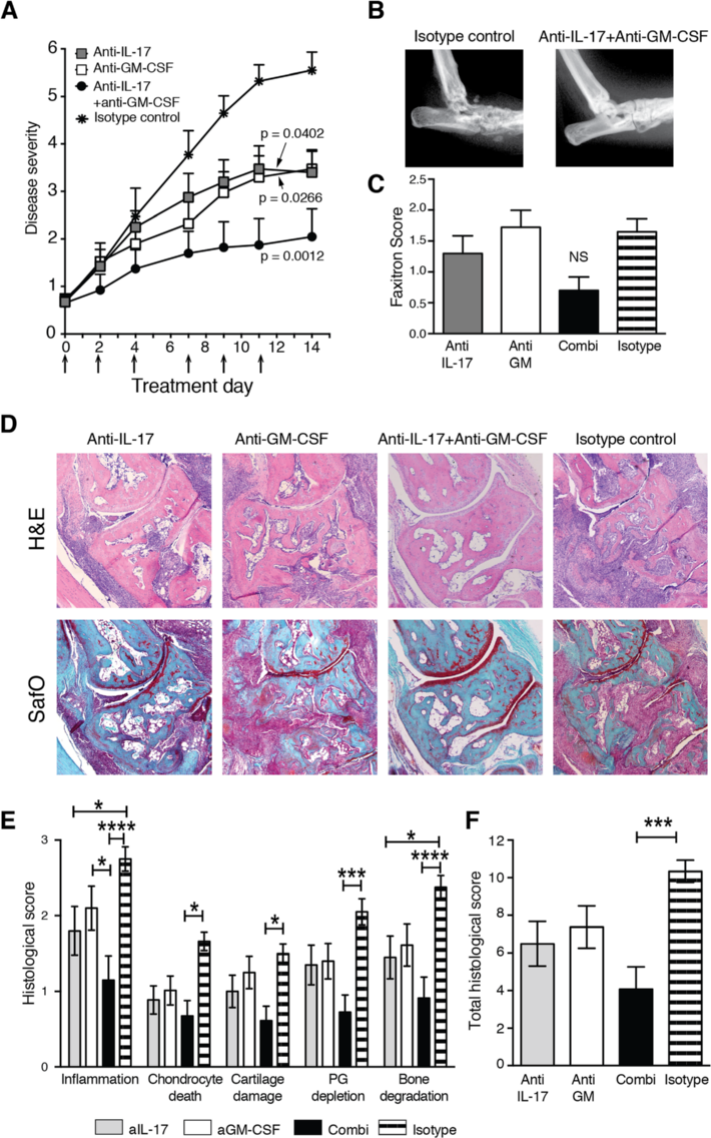
Neutralisation of interleukin (IL)-17 and granulocyte macrophage colony-stimulating factor (GM-CSF) during established collagen-induced arthritis (CIA). **a** Macroscopic disease scores followed over time from day 21 (day 0 of treatment). *n* = 10 mice/group. *Arrows* indicate times of antibody treatment. *p* values were calculated by two-way analysis of variance (ANOVA) after calculation of the area under the curve. **b** Endpoint X-ray analysis of ankle joints after CIA. Representative ankle joint shown for the isotype control and the anti-IL-17 + anti-GM-CSF groups. **c** Pooled endpoint X-ray analysis of ankle joints scored for bone damage [*n* = 20 joints/group; mean ± standard error of the mean (SEM)]. **d** Histological analysis of ankle joints. Original magnification, ×50. **e** Detailed histological scores of the ankle joints after 14 days of treatment. *n* = 20 joints/group. **f** Total histological scores. *n* = 20 joints/group. Mean ± SEM. *p < 0.05; ***p < 0.001; ****p < 0.0001, two-way ANOVA and Bonferroni’s test for multiple comparisons. *H&E* haematoxylin and eosin, *NS* not significant, *PG* proteoglycan, *SafO* safranin O stain

CIA was induced, and antibody treatment was started from the onset of disease. Mice that received the control antibody developed severe arthritis, reaching a macroscopic disease severity score of 5.55 ± 0.38 after 2 weeks of treatment (Fig. 1a). In contrast, a significant reduction in arthritis severity was observed after treatment with anti-IL-17 or anti-GM-CSF, resulting in endpoint scores of 3.4 ± 0.4 and 3.5 ± 0.4, respectively. Combination treatment completely ameliorated disease progression and resulted in a score of 2.0 ± 0.6 after 2 weeks of treatment. These effects were dose-dependent, with suboptimal combination therapy (30 μg of anti-IL-17 + 100 μg of anti-GM-CSF) reaching an end score of 3.9 ± 0.5, and suboptimal therapy with either antibody alone, resulting in end scores of 4.9 ± 0.4 for anti-GM-CSF and 3.5 ± 0.4 for anti-IL-17, respectively (data not shown). This result illustrates the additive beneficial effect of combined IL-17/GM-CSF neutralisation.

### Combined neutralisation of GM-CSF and IL-17 protects against bone and cartilage damage

Ankle joints were subjected to X-ray analysis to visualise bone destruction and changes in joint morphology (Fig. 1b, c). Bone damage in the small joints of the ankle and foot was observed in animals treated with anti-IL-17 or anti-GM-CSF alone (Fig. 1c), consistent with the macroscopic arthritis scores. Mice treated with the control antibody showed severe distortion of joint morphology and extensive bone damage (Fig. 1b, c). In contrast, most animals treated with a combination of anti-IL-17 and anti-GM-CSF showed near-normal joint morphology and minor or no bone degradation, although overall the average Faxitron scores were not significantly different between the groups (Fig. 1b, c). Other features of joint pathology were scored by histology on H&E- and SafO-stained, paraffin-embedded sections (Fig. 1d, e, f). Combination therapy resulted in a marked reduction of each feature scored (Fig. 1e) and was more effective than treatment with the separate antibodies (Fig. 1e, f).

### Differential local and systemic effects on inflammatory mediators by blockade of IL-17 or GM-CSF

**Fig. 2:**
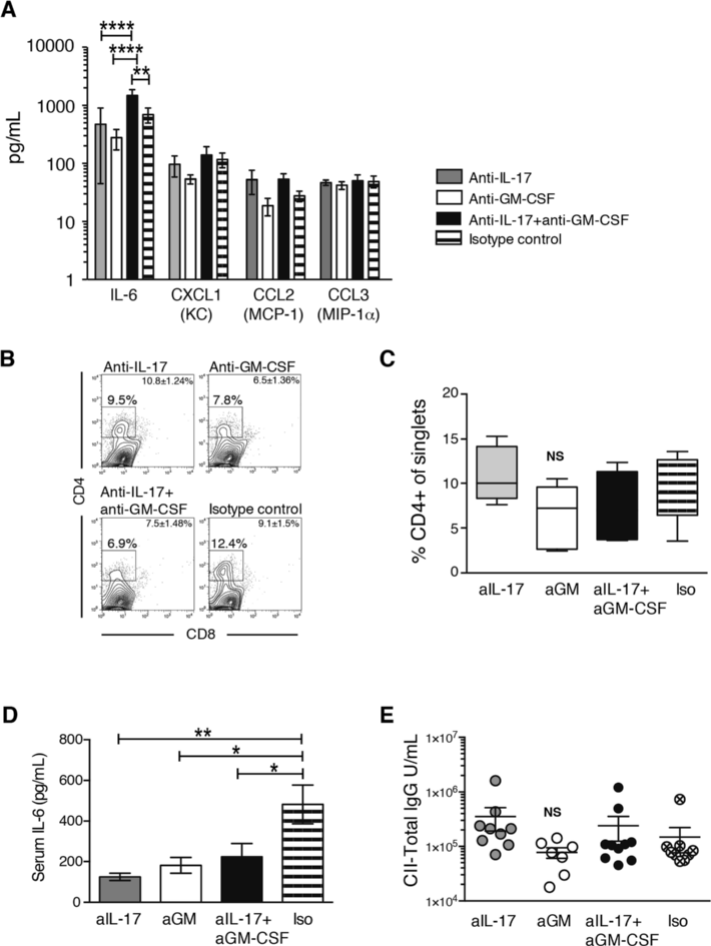
Cytokines in synovial washouts and serum and flow cytometric analysis of synovium and serum immunoglobulin G (IgG) after 14 days of interleukin (IL)-17 and granulocyte macrophage colony-stimulating factor (GM-CSF) blockade. **a** Luminex analysis of cytokines and chemokines in joint washouts (*n* = 6 mice per group). **b** Flow cytometric analysis of phorbol 12-myristate 13-acetate/ionomycin-stimulated synovial tissue stained with monoclonal antibodies for CD4 and CD8 after 14 days of treatment. The gate depicts the percentage of CD4^+^ cells of single synoviocytes. Plots shown are representative of *n* = 6 joints/group. Mean ± standard error of the mean (SEM) for each group is given in the fluorescence-activated cell sorting plot. **c** Summary graph for the flow cytometric analysis of synovial tissue. *Box* depicts 25th to 75th percentiles. *Line* depicts median. *Whiskers* depict minimal to maximal values. *n* = 6 mice per group. **d** IL-6 levels in serum measured by Luminex assay. *n* = 10 mice/group. Mean ± SEM. **e** Total collagen-specific immunoglobulin G (IgG) in serum. *n* = 7–10 mice/group. *p < 0.05; **p < 0.01; analysis of variance followed by Bonferroni’s test for multiple comparisons. *CCL* CC chemokine ligand, *CII* type II collagen, *CXCL* chemokine (C-X-C motif) ligand, *KC* keratinocyte, *MCP* monocyte chemoattractant protein, *MIP* macrophage inflammatory protein, *NS* not significant

Local production of cytokines and chemokines was measured in joint washouts. The major cytokines produced by arthritic synoviocytes were IL-6, chemokine (C-X-C motif) ligand 1 (CXCL1), CC chemokine ligand 2 (CCL2) and CCL3 (Fig. 2a). The production of IL-6 was not affected in mice treated with anti-IL-17 or anti-GM-CSF alone; however, combination treatment led to a significant increase in this cytokine compared with the other treatment groups (Fig. 2a). This is most likely due to the reduced influx of IL-6 receptor-expressing naive CD4^+^ T cells and the absence of inflammatory cells and osteoclasts in the joints of these mice, resulting in reduced consumption of IL-6 [4, 28]. Production of CXCL1, CCL2 and CCL3 was not affected by antibody treatment. In agreement with earlier studies, tumour necrosis factor (TNF) was not detected in the joint washouts [29, 30] (data not shown). Synovial cells were also analysed by flow cytometry to detect infiltrating CD4^+^ T cells. Anti-GM-CSF treatment and combination treatment resulted in a slight reduction of infiltrating CD4^+^ T cells. This effect was not observed in the mice treated with anti-IL-17 (Fig. 2b, c).

The systemic effect of antibody treatment was determined by analysis of serum cytokines. The level of IL-6 was significantly decreased in the antibody-treated animals, which is in agreement with recent data from clinical trials of GM-CSF inhibitors in RA [31] and previous data from our laboratory [12]. There was no difference between treatment groups (Fig. 2d). No other arthritis-related cytokines [IL-1β, TNF, IL-17, interferon-γ, IL-4, CCL5 (also called *RANTES*, for regulated on activation, normal T cell expressed and secreted) and GM-CSF] could be detected in the serum (data not shown). To assess the effect of GM-CSF or IL-17 blockade on the production of anti-collagen antibodies, collagen-specific IgG was measured in serum. A reduction in total CII-specific IgG was seen in the mice receiving anti-GM-CSF treatment, but it did not reach statistical significance (Fig. 2e).

### Neutralisation of granulocyte macrophage colony-stimulating factor, but not interleukin-17, inhibits production of matrix metalloproteinases

Cartilage and bone degradation are partly mediated by matrix metalloproteinases (MMPs) and alarmins, such as S100A8 [32, 33] and tissue inhibitors of metalloproteinase (TIMPs) [32]. The synovial expression of these and several T cell and DC/macrophage-related mediators was determined by qPCR (Table 1). We found a profound reduction in MMPs after GM-CSF neutralisation and a decrease in Th17-related factors GM-CSF, RORγt and IL-21. IL-17 mRNA was not detected in the anti-GM-CSF samples, but, interestingly, the expression of IL-23 was not different from that in the control group. Although anti-IL-17 treatment had a clear effect on joint damage, it was not reflected in the levels of MMP or TIMP mRNA in the joints of these mice. However, we also found a reduction in the level of the Th17-related factors RORγt, IL-21 and IL-23. Combination therapy led to an overall downregulation of MMPs. The effect on TIMP expression was modest in all treatment groups. Interestingly, IL-1β and IL-23 were upregulated after combined neutralisation (Table 1). This is most likely due to a reduction in consumption of these factors, owing to the lack of infiltrating inflammatory cells.

**Table 1:** Quantitative PCR of synovial tissue after 2 weeks of antibody treatment during collagen-induced arthritis (fold increase over isotype control-treated samples (2^−ΔΔCt^)

		Gene	Anti-IL-17	Anti-GM-CSF	Anti-IL-17 + anti-GM-CSF
Damaging	Proinflammatory T cell factors	*IL-17A*	1.63 ± 0.56	not detected	1.48 ± 1.0
		*GM-CSF*	1.12 ± 0.73	0.55 ± 0.54	0.55 ± 0.2
		*RORγt*	5.06 ± 2.94^a,b^	0.68 ± 0.30^c^	1.68 ± 0.96^c^
		*IL-21*	2.30 ± 1.14	0.88 ± 0.61	0.71 ± 0.35
		*RANKL*	0.97 ± 0.49	0.36 ± 0.19	0.80 ± 0.56
	Matrix metalloproteinases	*MMP3*	0.87 ± 0.45	0.24 ± 0.10	0.51 ± 0.26
		*MMP9*	1.18 ± 0.71	0.36 ± 0.18	0.75 ± 0.42
		*MMP13*	0.89 ± 0.38	0.44 ± 0.18	0.60 ± 0.31
		*MMP14*	1.43 ± 0.55	0.58 ± 0.22	0.74 ± 0.32
		*ADAMTS5*	1.34 ± 0.67	0.43 ± 0.24	0.89 ± 0.50
	Alarmin	*S100A8*	1.82 ± 0.93	0.56 ± 0.23	1.08 ± 0.58
	Pro-inflammatory DC/Mϕ-derived	*IL-1β*	1.09 ± 0.44	0.85 ± 0.40	1.67 ± 1.03
		*IL-23*	0.45 ± 0.19	1.02 ± 0.56	1.71 ± 0.89
Protective	Anti-inflammatory T cell factor	*IL-10*	1.53 ± 0.81	0.62 ± 0.38	0.71 ± 0.35
	Matrix metalloproteinase inhibitors	*TIMP1*	1.31 ± 0.56	0.48 ± 0.23	0.85 ± 0.42
		*TIMP2*	1.05 ± 0.39	0.50 ± 0.18	0.58 ± 0.25
		*TIMP3*	0.77 ± 0.09	0.63 ± 0.15	0.70 ± 0.09
		*TIMP4*	0.79 ± 0.19	0.97 ± 0.19	0.91 ± 0.22
	Th1/Th2 transcription factors	*T-bet*	1.75 ± 0.53	0.77 ± 0.11	0.84 ± 0.18
		*GATA3*	1.87 ± 0.71	0.67 ± 0.27	0.69 ± 0.20

*GM-CSF* granulocyte macrophage colony-stimulating factor, *IL* interleukin, *Th* helper T cell

*n* = 6 mice/treatment group

^a^ *p* ≤ 0.001 vs. anti-IL-17 + anti-GM-CSF (two-way analysis of variance and Bonferroni’s multiple-comparisons test)

^b^ *p* ≤ 0.0001 vs. anti-GM-CSF

^c^ *p* ≤ 0.0001 vs. anti-IL-17

### Overexpression of GM-CSF and IL-17 in the knee joint leads to joint destruction

**Fig. 3:**
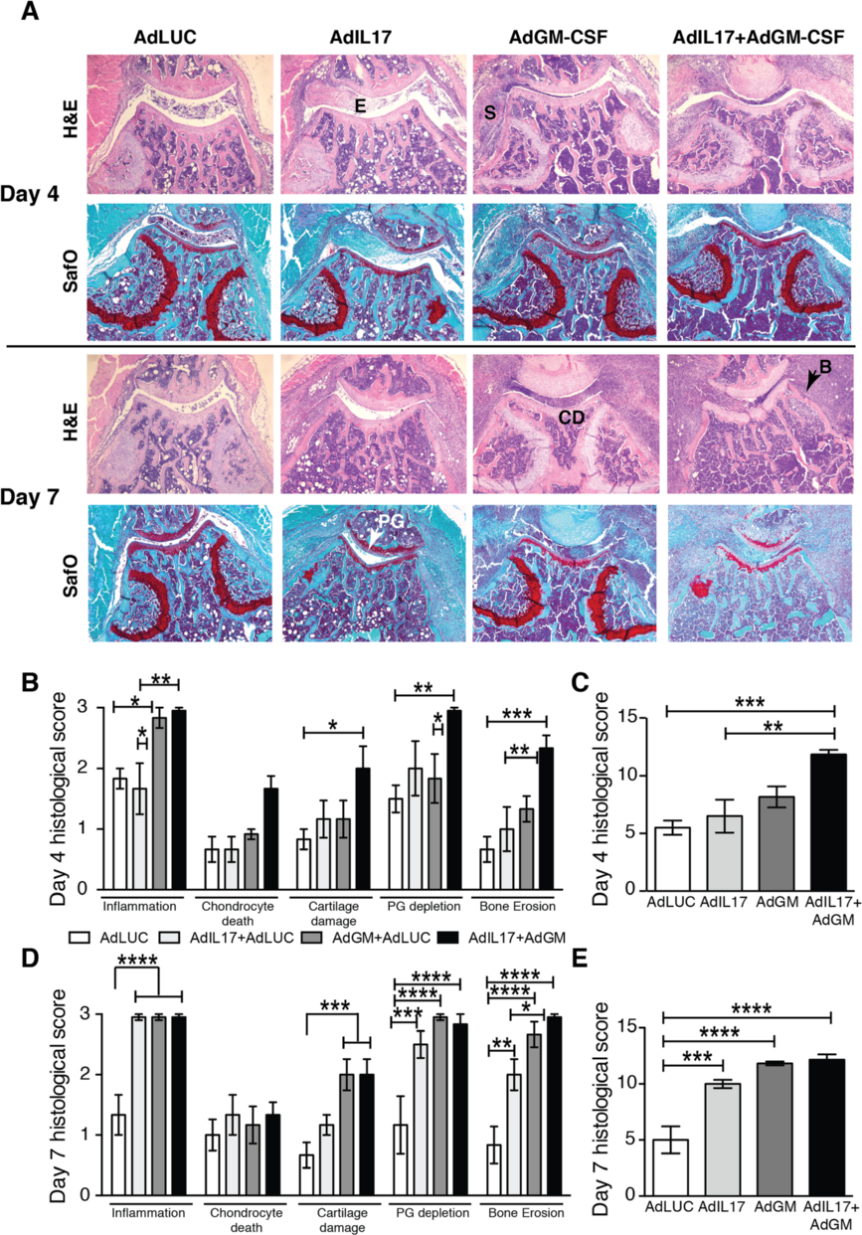
Histological analysis of joint damage after intraarticular injection of adenoviral vectors for interleukin (IL)-17 and granulocyte macrophage colony-stimulating factor (GM-CSF). **a** Haematoxylin and eosin (H&E)- and Safranin O (SafO)-stained sections of knee joints on days 4 and 7 after adenoviral transfer. *E* exudate, *S* synovitis, *CD* cartilage damage, *B* bone erosion, *PG* proteoglycan depletion. Representative sections are shown from *n* = 6 joints/group. **b** Individual and **c** total histological scores for day 4 after adenoviral transfer. **d** Individual and **e** total histological scores for day 7 after adenoviral transfer. Mean ± standard error of the mean for *n* = 6 joints per group. *p < 0.05; **p < 0.01; ***p < 0.001; ****p < 0.0001; analysis of variance followed by Bonferroni’s test for multiple comparisons

Our second approach to studying the IL-17–GM-CSF interaction was local overexpression in naive murine knee joints. Injection with Ad-IL-17 caused a mild inflammatory reaction in the joint on day 4, which increased to severe on day 7, when moderate to severe bone erosion and PG depletion were apparent (Fig. 3a–e). In contrast, Ad-GM-CSF injection led to severe synovitis and exudate on day 4, with increased bone erosion, PG depletion and cartilage damage on day 7. Injection with Ad-IL-17 + Ad-GM-CSF caused accelerated joint damage, with severe inflammation, bone erosion and PG depletion on day 4. Normal joint architecture was lost by day 7 owing to extensive inflammation (Fig. 3a), which suggested additive effects of these cytokines. Mice that received injections with the control vector Ad-LUC showed mild inflammation early after injection, but this resolved within 7 days.

### Combined expression of GM-CSF and IL-17 induces CXCL1, IL-6, IL-23, RANKL and MMPs in synovial tissue

**Fig. 4:**
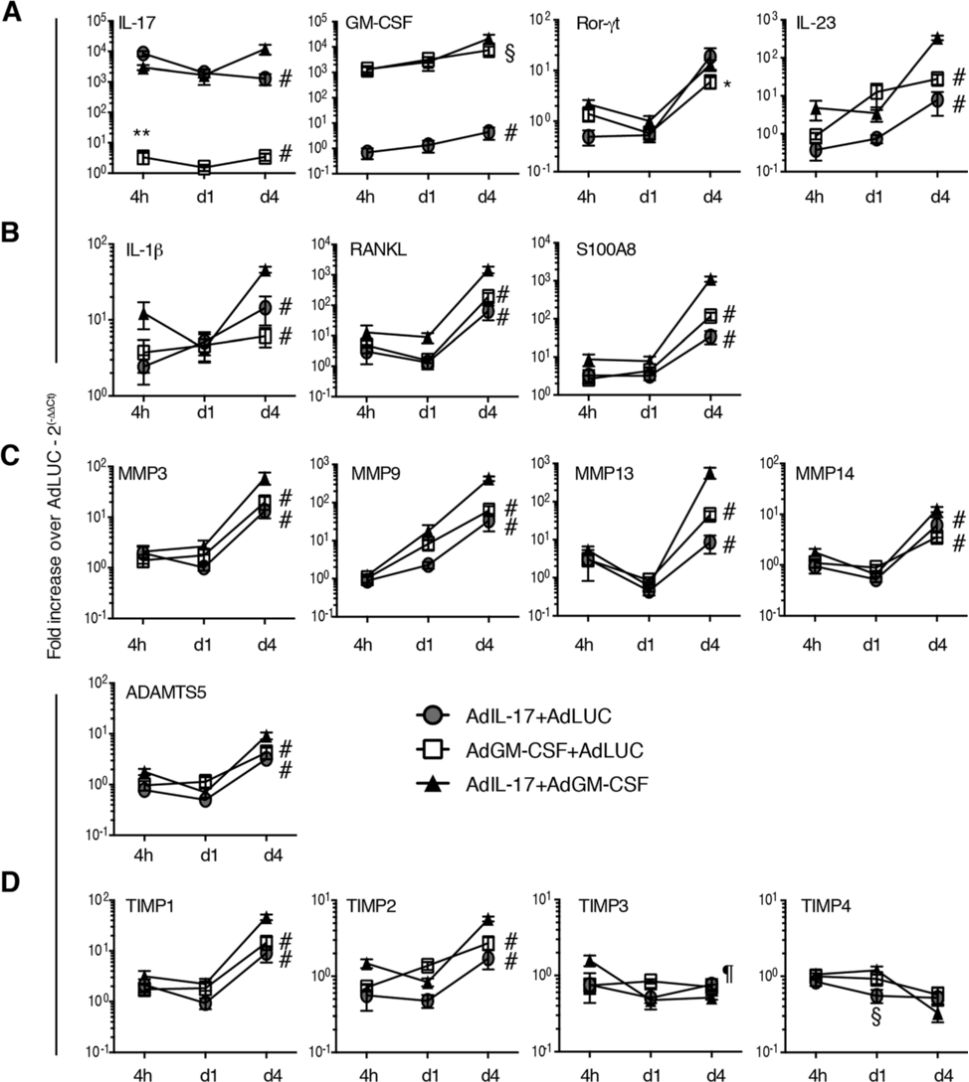
Quantitative PCR analysis of inflammatory and anti-inflammatory mediators in synovial tissue after adenoviral transfer into the knee joint. **a** Interleukin (IL)-17, granulocyte macrophage colony-stimulating factor (GM-CSF), RAR-related orphan receptor γt (RORγt) and IL-23 expression in synovial tissue determined at 4 h, day 1 and day 4 after adenoviral transfer. **b** Proinflammatory mediators IL-1β, receptor activator of nuclear factor κB ligand (RANKL) and S100A8 expression in synovial tissue determined at 4 h, day 1 and day 4 after adenoviral transfer. **c** Matrix metalloproteinase (MMP) expression in synovial tissue determined at 4 h, day 1 and day 4 after adenoviral transfer. **d** Expression of MMP inhibitors in synovial tissue determined at 4 h, day 1 and day 4 after adenoviral transfer. *n* = 6 joints/group. Mean ± standard error of the mean. *p < 0.05, **p < 0.01 vs. Ad-IL-17; ^§^ *p* < 0.05, ^¶^ *p* < 0.001, ^#^ *p* < 0.0001 vs. Ad-IL-17 + Ad-GM-CSF; analysis of variance followed by Bonferroni’s test for multiple comparisons. *ADAMTS5* a disintegrin and metalloproteinase with thrombospondin motifs, *TF* transcription factor, *TIMP* tissue inhibitor of matrix metalloproteinase

**Fig. 5:**
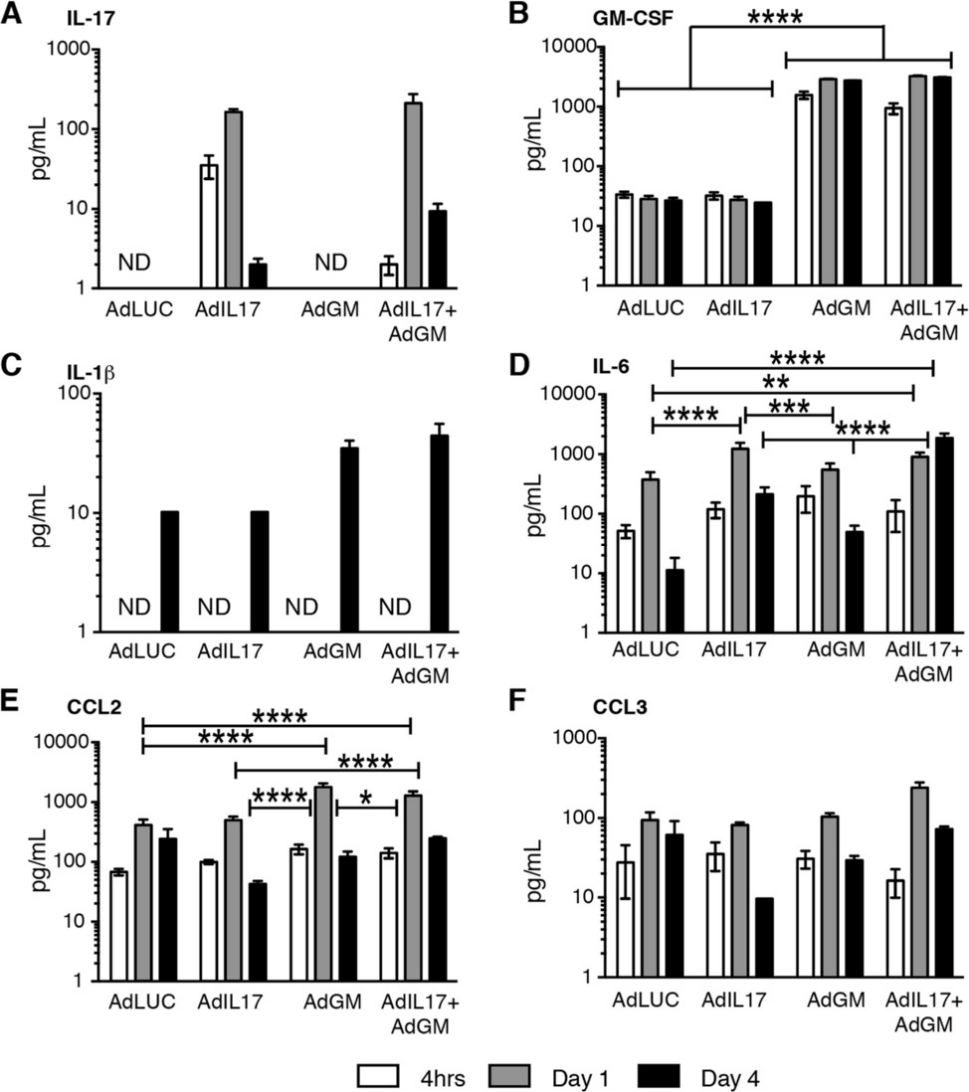
Cytokine and chemokine analysis of joint washouts after adenoviral transfer into the knee joint*.* Luminex analysis for cytokines and chemokines at 4 h, 1 day and 4 days after adenoviral transfer. **a** Interleukin (IL)-17. **b** Granulocyte macrophage colony-stimulating factor (GM-CSF). **c** IL-1β. **d** IL-6. **e** CC chemokine ligand (CCL2). **f** CCL3. *n* = 6 mice per group. Mean ± standard error of the mean. *ND* not detected. *p < 0.05; **p < 0.01; ***p < 0.001; ****p < 0.0001; analysis of variance followed by Bonferroni’s test for multiple comparisons

The expression of inflammatory cytokines, RORγt and MMPs was measured in synovial tissue by qPCR and Luminex analysis at 4 h, 1 day and 4 days after adenoviral transfer (Figs. 4 and 5). Increased transcription of IL-17A and GM-CSF in the mice transferred with the respective adenoviral vectors was confirmed by qPCR (Fig. 4a). Transcription levels were similar between the two cytokines (measuring a 10^3^ to 10^4^ increase over the control group transfected with Ad-LUC), and they remained at this level for at least 4 days after transfer. IL-17 mRNA began to decrease at day 4 in the mice transfected with Ad-IL-17 only; in mice that received both Ad-IL-17 and Ad-GM-CSF, transcription remained high. We did not detect any synergism at the transcriptional level between the cytokines in the mice injected with both vectors. Interestingly, the transcription of RORγt was increased in all groups on day 4, suggesting that Th17 differentiation in the joints was induced by the presence of IL-17 as well as GM-CSF (Fig. 4a). IL-23 increased on day 4 for the Ad-IL-17 group (Fig. 4a), but overexpression of GM-CSF induced IL-23 production earlier, in keeping with the relationship between GM-CSF and IL-23 in autoimmune inflammation [18]. Interestingly, combined overexpression induced an impressive increase in IL-23 on day 4, suggestive of a synergistic relationship (Fig. 4b). Although IL-17 or GM-CSF alone induced increased IL-1β, receptor activator of nuclear factor κB ligand (RANKL) and MMPs, the combination additionally led to increased production of RANKL, MMP9, MMP13 and S100A8 (Fig. 4b, c). Expression of TIMP1 and TIMP2, but not of TIMP3 and TIMP4, was increased in all groups (Fig. 4d).

Protein levels of IL-17 and GM-CSF were increased in joint washouts after transfection with the respective vector or with both vectors (Fig. 5). Interestingly, although the mRNA levels of IL-17 and GM-CSF were similar (Fig. 4), we observed a 10-fold increase in GM-CSF over IL-17 at the protein level, suggestive of increased translation, increased half-life or decreased consumption GM-CSF. The effect of combined overexpression was most noticeable in the production of IL-6, which showed a 10-fold increase over the mice treated with Ad-IL-17 or Ad-GM-CSF alone on day 4 (Fig. 5).

## Discussion

GM-CSF has recently been identified as a key player in Th17-mediated inflammation [16, 17, 23, 24]. Both GM-CSF and IL-17 are produced by T cells and are critical mediators of experimental arthritis [8, 13, 19, 23, 34, 35]. GM-CSF is essential for the pathogenicity of Th17 cells and for the CCR4-dependent production of IL-23 by DCs in EAE [16–18]. GM-CSF production by T cells is regulated by the transcription factor NF-κB1 [23], as well as by RORγt [16]. T cells lack GM-CSF receptors [36], so the effect of T cell–derived GM-CSF on Th17-mediated inflammation occurs via enhancement of IL-6 and IL-23 production from antigen-presenting cells [17]. IL-6 and IL-23 enhance GM-CSF production by T cells by binding to the respective receptors, creating a positive feedback loop [17]. In addition, IL-6 induces Th17 differentiation in combination with IL-23 and tumour growth factor β, creating a second feedback loop [37]. The close relationship between GM-CSF and IL-17 in inflammatory conditions suggests that therapeutic neutralisation of one molecule might affect both molecules. Indeed, prophylactic blockade of GM-CSF in IL-17 receptor-deficient mice improved disease outcome in a model of chronic relapsing arthritis [38]. The use of prophylactic treatment and knockout mice in that study does limit its clinical relevance, however.

In the present study, we show that combined therapeutic inhibition of IL-17 and GM-CSF significantly improves the outcome of CIA in wild type mice over inhibition of these cytokines separately by targeting different components of Th17-mediated joint inflammation. IL-17 mediates chondrocyte metabolism and can induce the expression of MMPs in synovial explants and fibroblasts [39, 40]. In primary cultures of human monocytes and macrophages, IL-17 stimulated the production of MMP9 [40]. In addition, IL-17 recruits neutrophils to the joint, inhibits the synthesis of chondrocyte PGs and induces the loss of PGs from mouse cartilage [11, 41]. We found that inhibition of IL-17 during established arthritis caused only a modest decrease in the local expression of MMP3, MMP13 and IL-23, but a marked reduction in systemic IL-6 levels, in accordance with previous reports [12]. TNF could not be detected in serum or joint washouts at the time of harvest, which confirms earlier data from our laboratory showing that TNF has an important role in the earlier stages of CIA but is less involved in the late chronic phase of disease [29, 30]. Anti-IL-17 did not prevent the influx of CD4^+^ T cells into the inflamed joints. This is in agreement with our study in the mBSA/IL-1β model of acute inflammatory arthritis, showing that it is the cytokine milieu in the inflamed joint that promotes Th17 differentiation from naive T cells in situ [8]. The beneficial effect of anti-IL-17 on joint pathology is therefore most likely due to the direct blockade of neutrophil influx, inhibition of Th17 differentiation and blockade of the Th17 feedback loop by reducing the levels of IL-23 and IL-6 in the synovium.

Anti-GM-CSF, either alone or in combination with anti-IL-17, had a profound effect on the synovial expression of MMPs, with effects ranging from a reduction in mRNA of 50 to 80 % (Table 1). This finding is in agreement with the fact that the main producers of MMPs are activated monocytes and macrophages [42], which depend on GM-CSF for proliferation and differentiation [43].

IL-6 was reduced systemically in the serum by both treatments; however, this reduction was not apparent in washouts of joints. In fact, combination treatment led to an increase in the local level of IL-6 (Fig. 2a), and synovial tissue showed increased expression of IL-1β and IL-23 in the absence of inflammation. The lack of activated T cells and the absence of inflammatory cells and osteoclasts could account for the increase in IL-6 measured via reduced consumption [4, 28]. The increase in IL-1β and IL-23 can be explained by increased local production of IL-17, induced by formation of IL-17-anti-IL-17 complexes or reduced consumption of these factors owing to the almost complete absence of inflammatory cells. In addition, mRNA levels were compared with those of animals receiving control antibodies. Inflammation in those mice progressed to peak severity, and many mice will be on the brink of disease remission owing to the loss of cartilage and a lack of collagen (Fig. 1c).

The role of IL-17 and GM-CSF was confirmed by overexpression of these cytokines in naive knee joints, adding an additional strategy to study local interactions. Intraarticularly injected adenoviral vectors preferentially transfect fibroblasts and synoviocytes [26]. Previous studies showed that overexpression of IL-17 in joints of mice during CIA increased bone erosion through the loss of the RANKL–osteoprotegerin balance [44] and that IL-17 induced MMPs only in the presence of TNF [29]. We found that the main effects of IL-17 overexpression were inflammation, PG depletion and bone erosion. We observed no significant differences between IL-17 and GM-CSF overexpression for any of the pathological features. By day 7, both IL-17 and GM-CSF caused maximum inflammation. Interestingly, although mRNA levels for IL-17 and GM-CSF were similar after transfection with the respective vectors (Fig. 4), the amount of GM-CSF protein measured in the Ad-GM-CSF joint washouts was increased up to 10-fold compared with IL-17 in the Ad-IL-17 injected joints. In addition, the level of GM-CSF remained high for the duration of the experiment, whereas IL-17 levels decreased after day 4 (Fig. 5). Whether this was due to (1) reduced IL-17 transcription by day 7, (2) a reduced half-life of IL-17 compared with GM-CSF or (3) an increased consumption of IL-17 is the subject of future investigation. Combined overexpression of IL-17 and GM-CSF led to accelerated and more severe pathology, suggestive of additive effects of these two cytokines. However, for the production of IL-23, RANKL and MMPs, there was a clear synergistic effect of IL-17 and GM-CSF. This was partly mirrored by a marked reduction of RANKL and MMPs after combined neutralisation, but, interestingly, neutralisation led to a local increase in IL-1β and IL-23. The absence of inflammation in the joints from animals treated with the combination therapy results in a markedly different composition of synovial cell populations, which is reflected in the qPCR results. These discrepancies may be resolved in future experiments either by standardising RNA levels to the expression of cell-specific markers (such as CD4) to correct for the difference in cell number or by analysing specific cell populations by flow cytometry rather than by qPCR.

## Conclusions

Overall, our results illustrate that complex interactions exist between IL-17 and GM-CSF during inflammation. Blockade of one of these cytokines can take away additive effects, but it does not by definition inhibit the other cytokine. Therefore, blocking both IL-17 and GM-CSF has an advantage over single-molecule inhibition. In view of the introduction of IL-17 and GM-CSF inhibitors in the clinic for autoinflammatory conditions such as RA and multiple sclerosis (recently reviewed in [4, 45, 46]), this is an important observation. Some patients do not respond to therapeutic inhibition, such as with TNF inhibitors [47]. On the basis of the additive beneficial effect of IL-17 and GM-CSF inhibition during CIA, combination therapy could greatly benefit this group of patients.

## Abbreviations

Ad: Adenovirus
ADAMTS: A disintegrin and metalloproteinase with thrombospondin motifs
ANOVA: Analysis of variance
CCL: CC chemokine ligand
CCR: CC chemokine receptor
CIA: Collagen-induced arthritis
CII: Type II collagen
Ct: Cycle threshold
CXCL: Chemokine (C-X-C motif) ligand
DC: Dendritic cell
EAE: Experimental autoimmune encephalitis
ffu: Focus forming unit
GM-CSF: Granulocyte macrophage colony-stimulating factor
H&E: Haematoxylin and eosin
IgG: Immunoglobulin G
IL: Interleukin
KC: Keratinocyte
LUC: Luciferase
mBSA: Methylated bovine serum albumin
MCP: Monocyte chemoattractant protein
MIP: Macrophage inflammatory protein
MMP: Matrix metalloproteinase
Mϕ: Macrophage
NS: Not significant
PG: Proteoglycan
qPCR: Quantitative real-time polymerase chain reaction
RA: Rheumatoid arthritis
RANKL: Receptor activator of nuclear factor κB ligand
RORγt: RAR-related orphan receptor gamma t
SafO: Safranin O
SEM: Standard error of the mean
TF: Transcription factor
Th: T helper
TIMP: Tissue inhibitor of matrix metalloproteinase
TNF: Tumour necrosis factor
